# Plant-LncPipe: a computational pipeline providing significant improvement in plant lncRNA identification

**DOI:** 10.1093/hr/uhae041

**Published:** 2024-02-08

**Authors:** Xue-Chan Tian, Zhao-Yang Chen, Shuai Nie, Tian-Le Shi, Xue-Mei Yan, Yu-Tao Bao, Zhi-Chao Li, Hai-Yao Ma, Kai-Hua Jia, Wei Zhao, Jian-Feng Mao

**Affiliations:** State Key Laboratory of Tree Genetics and Breeding, National Engineering Research Center of Tree Breeding and Ecological Restoration, Beijing Advanced Innovation Center for Tree Breeding by Molecular Design, National Engineering Laboratory for Tree Breeding, Key Laboratory of Genetics and Breeding in Forest Trees and Ornamental Plants, Ministry of Education, College of Biological Sciences and Technology, Beijing Forestry University, Beijing 100083, China; State Key Laboratory of Tree Genetics and Breeding, National Engineering Research Center of Tree Breeding and Ecological Restoration, Beijing Advanced Innovation Center for Tree Breeding by Molecular Design, National Engineering Laboratory for Tree Breeding, Key Laboratory of Genetics and Breeding in Forest Trees and Ornamental Plants, Ministry of Education, College of Biological Sciences and Technology, Beijing Forestry University, Beijing 100083, China; Rice Research Institute, Guangdong Academy of Agricultural Sciences & Key Laboratory of Genetics and Breeding of High Quality Rice in Southern China (Co-construction by Ministry and Province), Ministry of Agriculture and Rural Affairs & Guangdong Key Laboratory of New Technology in Rice Breeding, Guangzhou 510640, China; State Key Laboratory of Tree Genetics and Breeding, National Engineering Research Center of Tree Breeding and Ecological Restoration, Beijing Advanced Innovation Center for Tree Breeding by Molecular Design, National Engineering Laboratory for Tree Breeding, Key Laboratory of Genetics and Breeding in Forest Trees and Ornamental Plants, Ministry of Education, College of Biological Sciences and Technology, Beijing Forestry University, Beijing 100083, China; State Key Laboratory of Tree Genetics and Breeding, National Engineering Research Center of Tree Breeding and Ecological Restoration, Beijing Advanced Innovation Center for Tree Breeding by Molecular Design, National Engineering Laboratory for Tree Breeding, Key Laboratory of Genetics and Breeding in Forest Trees and Ornamental Plants, Ministry of Education, College of Biological Sciences and Technology, Beijing Forestry University, Beijing 100083, China; State Key Laboratory of Tree Genetics and Breeding, National Engineering Research Center of Tree Breeding and Ecological Restoration, Beijing Advanced Innovation Center for Tree Breeding by Molecular Design, National Engineering Laboratory for Tree Breeding, Key Laboratory of Genetics and Breeding in Forest Trees and Ornamental Plants, Ministry of Education, College of Biological Sciences and Technology, Beijing Forestry University, Beijing 100083, China; State Key Laboratory of Tree Genetics and Breeding, National Engineering Research Center of Tree Breeding and Ecological Restoration, Beijing Advanced Innovation Center for Tree Breeding by Molecular Design, National Engineering Laboratory for Tree Breeding, Key Laboratory of Genetics and Breeding in Forest Trees and Ornamental Plants, Ministry of Education, College of Biological Sciences and Technology, Beijing Forestry University, Beijing 100083, China; State Key Laboratory of Tree Genetics and Breeding, National Engineering Research Center of Tree Breeding and Ecological Restoration, Beijing Advanced Innovation Center for Tree Breeding by Molecular Design, National Engineering Laboratory for Tree Breeding, Key Laboratory of Genetics and Breeding in Forest Trees and Ornamental Plants, Ministry of Education, College of Biological Sciences and Technology, Beijing Forestry University, Beijing 100083, China; Key Laboratory of Crop Genetic Improvement & Ecology and Physiology, Institute of Crop Germplasm Resources, Shandong Academy of Agricultural Sciences, Jinan 250100, China; Department of Plant Physiology, Umeå Plant Science Centre (UPSC), Umeå University, Umeå 90187, Sweden; State Key Laboratory of Tree Genetics and Breeding, National Engineering Research Center of Tree Breeding and Ecological Restoration, Beijing Advanced Innovation Center for Tree Breeding by Molecular Design, National Engineering Laboratory for Tree Breeding, Key Laboratory of Genetics and Breeding in Forest Trees and Ornamental Plants, Ministry of Education, College of Biological Sciences and Technology, Beijing Forestry University, Beijing 100083, China; Department of Plant Physiology, Umeå Plant Science Centre (UPSC), Umeå University, Umeå 90187, Sweden

## Abstract

Long non-coding RNAs (lncRNAs) play essential roles in various biological processes, such as chromatin remodeling, post-transcriptional regulation, and epigenetic modifications. Despite their critical functions in regulating plant growth, root development, and seed dormancy, the identification of plant lncRNAs remains a challenge due to the scarcity of specific and extensively tested identification methods. Most mainstream machine learning-based methods used for plant lncRNA identification were initially developed using human or other animal datasets, and their accuracy and effectiveness in predicting plant lncRNAs have not been fully evaluated or exploited. To overcome this limitation, we retrained several models, including CPAT, PLEK, and LncFinder, using plant datasets and compared their performance with mainstream lncRNA prediction tools such as CPC2, CNCI, RNAplonc, and LncADeep. Retraining these models significantly improved their performance, and two of the retrained models, LncFinder-plant and CPAT-plant, alongside their ensemble, emerged as the most suitable tools for plant lncRNA identification. This underscores the importance of model retraining in tackling the challenges associated with plant lncRNA identification. Finally, we developed a pipeline (Plant-LncPipe) that incorporates an ensemble of the two best-performing models and covers the entire data analysis process, including reads mapping, transcript assembly, lncRNA identification, classification, and origin, for the efficient identification of lncRNAs in plants. The pipeline, Plant-LncPipe, is available at: https://github.com/xuechantian/Plant-LncRNA-pipline.

## Introduction

Long non-coding RNAs (lncRNAs) are RNA molecules longer than 200 nucleotides in length and do not encode proteins. Although they were initially thought to be transcriptional noise, recent research has uncovered that lncRNAs play crucial roles in a variety of biological processes, such as transcriptional regulation, chromatin remodeling, and RNA splicing [1, 2]. In plants, lncRNAs have been found to regulate plant growth and development, chromatin modification, and responses to environmental stresses [3, 4]. Consequently, the identification of lncRNAs in plants is vital for unraveling the molecular mechanisms underlying numerous biological processes. Although several lncRNA prediction methods currently exist, most of them were developed using human or other animal datasets, making it challenging to accurately identify lncRNAs in plants. Despite these challenges, two main strategies have recently been employed to identify and annotate lncRNAs in plant species. One strategy involves the use of comparative genomics to identify conserved non-coding regions [5]. Another strategy employs machine learning algorithms to classify transcripts based on their sequence and structural features, evidenced by the development of computational tools for lncRNA identification, such as CPC2 [6], CNCI [7], CPAT [8], PLEK [9], LncADeep [10], and LncFinder [11]. These tools utilize machine learning algorithms based on features such as open reading frame (ORF) length, codon usage bias, and support vector machine *k*-mer frequencies to distinguish lncRNAs from protein-coding RNAs. CPC2, CNCI, LncFinder, and PLEK use a support vector machine (SVM) model to calculate the coding potential of transcripts, while CPAT employs a logistic regression model to differentiate coding and non-coding transcripts. LncADeep is a computational tool that uses deep learning algorithms for lncRNA identification [10].

Nonetheless, most of these tools were trained using human or other animal datasets, and the potential benefits of retraining with plant data have not been explored. While there are specialized software tools for plant lncRNA identification, like RNAplonc [12], PLncPRO [13], and CREMA [14], due to the limited range of users used, their capacity to accurately identify the majority of plant lncRNAs still requires further validation. As a result, enhancing the accuracy and comprehensiveness of plant lncRNA prediction and determining the most fitting plant lncRNA prediction software necessitates further investigation. In addition, retraining mainstream models and developing ensemble methods are valuable to further improve plant lncRNA prediction.

**Table 1:** . Number of lncRNAs and mRNAs in the training set for model construction.

Dataset	Species	lncRNA (GreeNC/PlanDB)		mRNA (Phytozome)
		High confidence	Validated	
Training	*Arabidopsis thaliana*	1697	149	1846
	*Cucumis sativus*	1792	13	1805
	*Glycine max*	1797	1	1798
	*Oryza sativa*	1770	40	1810
	*Solanum lycopersicum*	1800	19	1819
	*Populus trichocarpa*	1784	18	1802

**Table 2:** Number of lncRNAs and mRNAs in the test set for model evaluation.

Test dataset	Species	lncRNA			mRNA (Phytozome/Ensembl Plants)
		CANTATAdb	GreeNC	PlncDB	
Angiosperms	*Ananas comosus*	3000	3000	3000	9000
	*Amborella trichopoda*	3000	3000	3000	9000
	*Arabidopsis thaliana*	3000	3000	3000	9000
	*Brachypodium distachyon*	3000	3000	3000	9000
	*Cucumis sativus*	1929	1929	1929	5787
	*Glycine max*	3000	3000	3000	9000
	*Manihot esculenta*	2655	2655	2655	7965
	*Medicago truncatula*	3000	3000	3000	9000
	*Musa acuminata*	2988	2988	2988	8964
	*Oryza sativa*	2600	2600	2600	7800
	*Populus trichocarpa*	3000	3000	3000	9000
	*Solanum lycopersicum*	3000	3000	3000	9000
	*Sorghum bicolor*	2600	2600	2600	7800
	*Vitis vinifera*	2974	2974	2974	8922
	*Zea mays*	3000	3000	3000	9000
Algae	*Chlamydomonas reinhardtii*	619	619	0	1238
	*Coccomyxa subellipsoidea*	668	668	0	1336
	*Micromonas pusilla*	640	640	0	1280
	*Volvox carteri*	1088	1088	0	2176
Bryophyte	*Physcomitrella patens*	5000	5000	0	10 000

In this study, we retrained the models of three widely used lncRNA identification methods, LncFinder, CPAT, and PLEK, with well-curated plant data and assessed their performance against the original models. Furthermore, we compared the retrained models with other popular lncRNA identification methods, including CPC2, CNCI, LncADeep, and RNAplonc. Based on the existing literature, RNAplonc outperforms other plant lncRNA identification software [12], so we chose it as the representative in our benchmarking. Our retrained models exhibited significant improvements, and the retrained versions of LncFinder and CPAT, LncFinder-plant and CPAT-plant, outperform other computational tools in plant lncRNA prediction. Ultimately, we implemented a pipeline (Plant-LncPipe) comprising these two models to carry out all essential steps in lncRNA identification and characterization.

## Results

### Retraining the CPAT, LncFinder, and PLEK models for plant lncRNA identification

**Figure 1:**
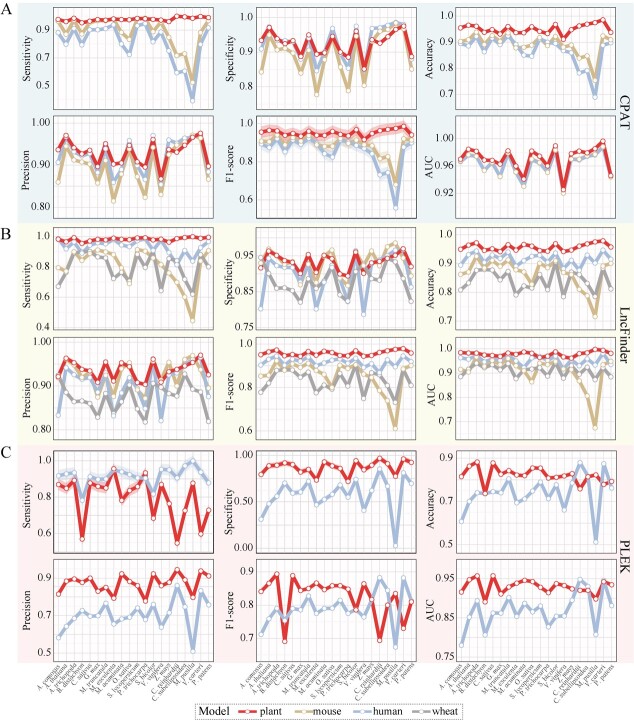
Evaluation of retrained models using data sets from 20 plant species. **A** Performance comparison of the retrained model (CPAT-plant) and the original models (CPAT-mouse and CPAT-human) of CPAT in terms of sensitivity, accuracy, precision, specificity, F1-score, and AUC. **B** Performance comparison of the retrained model (LncFinder-plant) with the original models (LncFinder-mouse, LncFinder-human, and LncFinder-wheat) of LncFinder in terms of sensitivity, accuracy, precision, specificity, F1-score, and AUC. **C** Performance comparison of the retrained model (PLEK-plant) and the original model (PLEK-human) of PLEK in terms of sensitivity, accuracy, precision, specificity, F1-score, and AUC.

**Figure 2:**
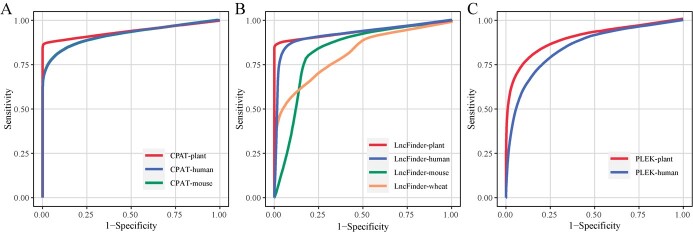
ROC curves of the retrained and original models on datasets from 20 plant species. **A** ROC curves of the retrained CPAT-plant model and its comparison with the original models for human and mouse. **B** ROC curves of the retrained LncFinder-plant model and comparison with the original models for human, mouse, and wheat. **C** ROC curves of the retrained PLEK-plant model and its comparison with the original model for human.

To enhance the quantity and accuracy of plant lncRNA identification, we retrained the models developed in LncFinder, CPAT, and PLEK using six plant datasets (Table 1) and evaluated their classification performance on 20 plant species, including 15 angiosperms, 4 algae, and 1 bryophyte (Table 2). We employed sensitivity, specificity, accuracy, precision, F1-score, and ROC–AUC metrics to assess the performance of these models. The results for CPAT indicated that the retrained model demonstrated significantly higher sensitivity, precision, accuracy, and F1-score compared with the original human and mouse models (Fig. 1A, Supplementary Data Tables S1 and S2). Moreover, the enhanced performance of the retrained CPAT model (CPAT-plant) is further underscored by the improved area ROC (receiver operating characteristic) curve and AUC (area under the curve) values in comparison with the original model (Figs 1A and 2A, Supplementary Data Fig. S1). Furthermore, 10-fold cross-validation of CPAT-plant on the training data demonstrates the robust optimization of CPAT for plant lncRNA identification through retraining (Supplementary Data Table S3).

Our results also demonstrate that the retrained LncFinder model (LncFinder-plant) outperforms the original models developed using mouse, wheat, and human training data. The LncFinder-plant model exhibits higher sensitivity, precision, accuracy, specificity, and F1-score on 20 test datasets when compared with these original models (Fig. 1B, Supplementary Data Tables S4 and S5). The ROC curve and AUC value of the LncFinder-plant model displayed enhanced performance compared with the default models (LncFinder-human, LncFinder-mouse, and LncFinder-wheat), suggesting a better trade-off between sensitivity (true positive rate) and specificity (false positive rate) (Figs 1B and 2B, Supplementary Data Fig. S2). The results of the 10-fold cross-validation analysis further support the superior classification performance of the LncFinder-plant model (Supplementary Data Table S6). Especially on algae, CPAT-plant and LncFinder were significantly higher in accuracy, sensitivity, and F1 score than the default models (Fig. 1A and B).

We next assessed the performance of the retrained PLEK model (PLEK-plant). Our findings revealed that, unlike CPAT-plant and LncFinder-plant, the sensitivity of the retrained PLEK model (PLEK-plant) decreased (Fig. 1C, Supplementary Data Tables S7 and S8). However, PLEK-plant exhibited enhanced specificity, precision, and accuracy (Fig. 1C). Moreover, the higher AUC value and better ROC of PLEK-plant also demonstrate that the performance of the retrained model is superior to the original (Figs 1C and 2C, Supplementary Data Fig. S3). Thus, although the sensitivity of PLEK-plant was diminished relative to the original model, its enhanced performance in other metrics suggests that the retrained model provides a more balanced and reliable approach for plant lncRNA identification.

### Benchmarking lncRNA identification tools

**Figure 3:**
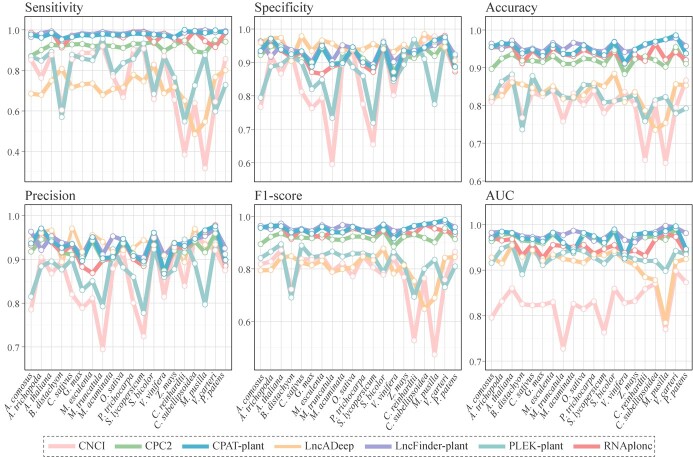
Performance evaluation of seven lncRNA identification tools in terms of sensitivity, accuracy, precision, specificity, F1-score, and AUC on datasets from 20 plant species.

**Figure 4:**
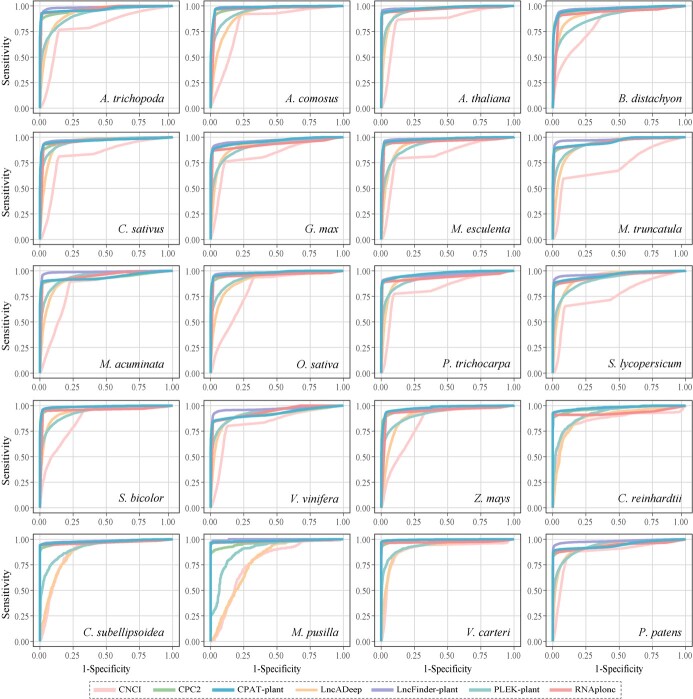
ROC curve of seven lncRNA identification methods on datasets from 20 plant species.

We conducted a comparative analysis of the retrained models’ performance against CNCI, CPC2, LncADeep, and RNAplonc using datasets from 20 plant species. Our results revealed that LncFinder-plant displayed the highest levels of sensitivity, accuracy, precision, F1-score, and AUC values, with CPAT-plant ranking as the second best performer in these evaluation metrics (Fig. 3A, Supplementary Data Tables S9–S11). Among the 20 test datasets, LncFinder-plant exhibited the highest performance on ROC curves, followed closely by CPAT-plant, indicating their superior performance compared with other software (Fig. 3B). Although the retrained model PLEK-plant exhibited improvement in predicting lncRNA, its performance was subpar compared with other methods (Figs 3 and 4), making it less competitive compared with CPAT-plant and LncFinder-plant. Despite its recent development and notable performance, RNAplonc falls behind both CPAT-plant and LncFinder-plant in terms of sensitivity, specificity, accuracy, F1-score, and ROC curve and AUC values. (Figs 3 and 4, Supplementary Data Table S11). While certain software tools display high specificity, such as LncADeep and CPC2, their overall efficacy in accurately identifying lncRNAs in plants is often limited due to lower scores in other crucial metrics (Fig. 3, Supplementary Data Table S11). Therefore, we recommend CPAT-plant and LncFinder-plant as the most effective tools for detecting plant non-coding RNAs, owing to their superior performance in various evaluation metrics.

### Reverse validation with animal data

To validate the suitability of the retrained plant models for CPAT and LncFinder, we performed a reverse validation using animal datasets (*Homo sapiens*, *Mus musculus*, and *Drosophila melanogaster*) as negative controls (Supplementary Data Table S12). For the CPAT model, we observed that the CPAT-plant model performed poorly on animal datasets compared with the models trained with mouse (CPAT-mouse) and human (CPAT-human) datasets (Supplementary Data Table S12). For instance, the CPAT-mouse model, when applied to mouse data, exhibits a sensitivity of 94.40%, a specificity of 97.71%, an accuracy of 96.04%, a precision of 97.63%, and an F1 score of 95.99%. Conversely, the CPAT-plant model shows comparatively lower performance metrics in plant data, with a sensitivity of 91.88%, a specificity of 96.69%, an accuracy of 94.27%, a precision of 96.52%, and an F1 score of 94.14% (Supplementary Data Table S12). Similar patterns were observed for the *D. melanogaster* and *H. sapiens* datasets. Additionally, the ROC curves and AUC values revealed that the original models consistently outperform the CPAT-plant model across the three animal datasets (Supplementary Data Fig. S4A, Supplementary Data Table S12), providing further evidence that the plant dataset is not suitable for animal lncRNA prediction. Likewise, LncFinder-plant also exhibited lower sensitivity, specificity, accuracy, precision, and F1-score for the animal datasets. For example, in the mouse dataset, the original LncFinder model (LncFinder-mouse) demonstrated higher sensitivity, specificity, accuracy, precision, and F1-score values of 94.99%, 96.73%, 95.87%, 96.68%, and 95.82% respectively, compared with the LncFinder-plant model, which had values of 91.45%, 95.53%, 93.50%, 95.34%, and 93.35% (Supplementary Data Table S13). The ROC curves and AUC values further supported the result that the retrained model, LncFinder-plant, is suboptimal for animal datasets (Supplementary Data Fig. S4B, Supplementary Data Table S13). Therefore, although these plant models demonstrate some effectiveness on animal datasets, their performance is not as strong as the original models that were specifically designed for animal data.

These reverse validation results provide compelling evidence that the plant-specific models, CPAT-plant and LncFinder-plant, are more suitable for lncRNA identification in plant datasets. Therefore, our study demonstrates the necessity for specialized models tailored to specific species and emphasizes the importance of using appropriate models for accurate lncRNA identification.

### An ensemble approach further improves performance of lncRNA prediction

Our results revealed that the retrained CPAT-plant and LncFinder-plant models were the top two performers in accurately predicting plant lncRNAs. Given that a single software may not provide sufficient stringency for lncRNA identification, we employed an ensemble approach incorporating both high-performing models, CPAT-plant and LncFinder-plant, to identify lncRNAs in plant species. The results demonstrated that the ensemble method exhibited higher precision across all 20 transcript datasets, consistently showcasing lower error rates (Supplementary Data Table S14). For example, the precision of lncRNA identification increased from 93.58% and 92.15% to 94.20% when implementing the ensemble method (Supplementary Data Table S14), resulting in a decrease in error rates from 6.42% and 7.58% to 5.80% for *Amborella trichopoda* compared with the utilization of CPAT-plant and LncFinder-plant individually (Supplementary Data Table S14). Therefore, our study demonstrated that employing an ensemble of CPAT-plant and LncFinder-plant is an effective and accurate approach for identifying plant lncRNAs. By reducing false positives of lncRNA identification, this ensemble method can generate more reliable results.

### Computational pipeline for lncRNA identification and characterization

**Figure 5:**
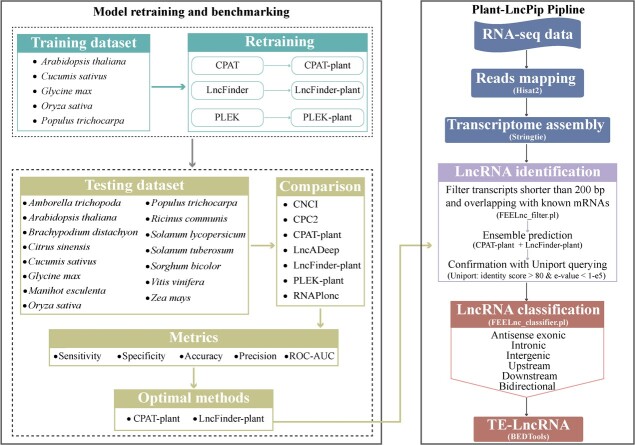
Workflow of the present study and the pipeline for lncRNA identification and characterization. The left panel illustrates our present workflow. The right panel depicts the process of a computational pipeline, Plant-LncPipe, which provides an ensemble method of lncRNA identification and key steps of lncRNA characterization.

We developed a pipeline (Plant-LncPipe) for the identification and characterization of plant lncRNAs by implementing key steps of lncRNA analysis: transcriptome alignment and assembly; lncRNA prediction; and lncRNA classification and origin (Fig. 5). We classified lncRNAs into six categories based on their genomic locations, e.g. intronic lncRNAs, intergenic lncRNAs, antisense exonic lncRNAs, upstream lncRNAs, downstream lncRNAs, and bidirectional lncRNAs (Fig. 5). In this pipeline, we defined upstream and downstream lncRNAs as those located within 2000 bp of the transcription start site and transcription termination site, respectively, but this range can be adjusted as per specific research requirements, such as extending to 50 kb [15]. This pipeline facilitates the easy classification of lncRNAs and provides insights into the diversity of lncRNAs in the genome. Moreover, the pipeline enables the analysis of intersections with transposable elements (TEs) within lncRNAs. Such analysis may yield new insights into the relationship between TEs and the origins of different categories of lncRNAs, considering that TEs are a major contributor to plant lncRNAs [16, 17].

## Discussion

Machine-learning algorithms have played an essential role in identifying and classifying lncRNAs from high-throughput RNA sequencing datasets. Several software tools, such as CPAT, LncFinder, CNCI, CPC2, and PLEK, have been developed for lncRNA identification with distinct training features and datasets. CPAT and PLEK were developed using logistic regression and SVM algorithms, respectively. CPAT predicts lncRNAs based on four features, i.e. maximum ORF length, ORF coverage, Fickett score, and codon usage bias, while PLEK employs an improved *k*-mer frequency distribution strategy to classify RNA sequences. LncFinder integrates features based on sequence intrinsic composition, structural information, and physicochemical properties, and uses an SVM algorithm for non-coding RNA identification. However, it is worth noting that most tools are primarily developed using human or other animal data for model retraining, while there is a lack of models trained with plant data. Given the differences in lncRNA features between plant and animal species [18, 19], retraining these models using plant datasets is critical to enhancing their accuracy and applicability in plant lncRNA identification.

In this study, we achieved significant improvement in predicting lncRNAs in plants by retraining models with plant transcriptome datasets. Our results showed that the retrained LncFinder-plant and CPAT-plant demonstrated significantly better performance in multiple metrics compared with their original versions. However, in the identification of plant lncRNAs using the LncFinder-wheat model, we found that performance was considerably subpar. This deficient performance of the wheat model in identifying plant lncRNAs can likely be ascribed to several specific causes. Primarily, the training dataset of this model has a high frequency of homologous genes, where ‘homologous’ is defined in this context as having an identity >80%. Specifically, amongst 4000 lncRNAs in the training set, 299 were homologous genes, and similarly, in the mRNA training set, 641 out of 4000 genes displayed homology. The excessive proportion of homologous genes may distort the model’s learning mechanism by associating certain features typically found in these genes with either lncRNAs or mRNAs. This could result in an elevated rate of false positives or negatives, thereby reducing the accuracy of the model’s predictive capabilities. Moreover, the substantial presence of homologous genes in the training data might induce overfitting during the training of the model. Consequently, this overfitting would restrict the model’s capacity to generalize effectively on novel data. Furthermore, the volume of training sets harnessed for wheat is insufficient and a single plant species may have affected its ability to adequately learn and predict lncRNA, thereby affecting its overall performance. A model trained on a comprehensive dataset encompassing multiple plant species is more likely to capture a wider range of lncRNA characteristics, thereby enhancing its ability to accurately identify lncRNAs in diverse plants.

The retrained PLEK-plant exhibits lower sensitivity, despite a noteworthy improvement in classification accuracy, precision, and F1-score compared with the original human model. These differences in performance improvement can be attributed to the choice of identification features used by each software. Both CPAT and LncFinder appear to have benefited from the model reconstruction as their algorithms rely on features or sequence information more closely related to the training dataset. CPAT employs a logistic regression model based on four sequence features, which could potentially be better adapted to the plant dataset following reconstruction. Similarly, LncFinder utilizes multiple sequence-derived features, which may have also led to improved performance after adapting to the plant dataset. In contrast, PLEK only employs a *k*-mer scheme for its classification. The decreased sensitivity of PLEK may result from its *k*-mer strategy being less sensitive to changes in the training dataset, rendering it incapable of capturing more specific sequence features of plant lncRNA.

We assessed the performance of the retrained models against that of other commonly used lncRNA identification tools, such as CNCI, CPC2, LncADeep, and RNAplonc. Our findings revealed that the retrained LncFinder-plant and CPAT-plant outperform all other lncRNA identification tools. This suggests that retraining using plant-specific data can significantly enhance plant lncRNA identification ability and accuracy. Furthermore, our results demonstrated that an ensemble of CPAT-plant and LncFinder-plant is a more effective approach for identifying lncRNAs in plants. The combined approach can be used as a reliable tool for identifying plant lncRNAs in a broad range of species and could potentially contribute to the discovery of novel lncRNAs. Additionally, we developed a pipeline (Plant-LncPipe) for identifying and characterizing plant lncRNAs. By using this pipeline, we can take advantage of the ensemble method (CPAT-plant + LncFinder-plant) in plant lncRNA identification, thus generating a more comprehensive understanding of the lncRNA landscape in plants and its involvement in diverse biological processes.

This study highlights the importance of model retraining. By utilizing comprehensive data, our retrained models can more accurately capture the intricacies of plant lncRNA identification, resulting in enhanced accuracy and reliability. Secondly, the development of a new model requires considerable resources, whereas model retraining leverages the computational infrastructure of mature software, significantly reducing development costs. Thirdly, retraining existing and mature lncRNA identification models ensures the preservation of prior knowledge. Previously established models, such as those created with CPAT and LncFinder, have undergone extensive testing, refinement, and validation. By retraining these models, the foundation of knowledge is augmented, enabling the development of an enhanced model for plant species without disregarding the accumulated expertise from previous developments. Researchers who are already familiar with the original software can readily adapt to the retrained models, thereby promoting widespread adoption and utilization of these enhanced models. In conclusion, this study contributes to the further advancement of more efficient and accurate tools for plant lncRNA prediction and characterization.

## Materials and methods

### Data collection

For model retraining, we meticulously selected data from six plant species: *Arabidopsis thaliana*, *Cucumis sativus*, *Glycine max*, *Oryza sativa*, *Populus trichocarpa*, and *Solanum lycopersicum* (Table 1). The lncRNA data were obtained from the GreeNC [20] and PlanDB [21] databases. The mRNA data were collected from the Phytozome [22] database. To evaluate the performance of our retrained models, we utilized an expanded test set of lncRNAs from 20 plant species (*Amborella trichopoda*, *Ananas comosus*, *A. thaliana*, *Brachypodium distachyon, C. sativus*, *G. max*, *Manihot esculenta*, *Medicago truncatula*, *Musa acuminata*, *O. sativa*, *P. trichocarpa*, *S. lycopersicum*, *Sorghum bicolor*, *Vitis vinifera*, *Zea mays*, *Chlamydomonas reinhardtii*, *Coccomyxa subellipsoidea*, *Micromonas pusilla*, *Volvox carteri*, and *Physcomitrella patens*) obtained from the CANTATAdb [23], GreeNC, and PlanDB database, as well as mRNAs from the Phytozome or Ensembl Plants [24] database (Table 2).

In order to avoid overfitting our training data, we removed redundant sequences with >80% identity using CD-HIT-EST (v4.8.1) [25]. In this context, lncRNAs were treated as the positive set, while mRNAs served as the negative set.

### Training and evaluation of lncRNA identification tools

LncFinder, CPAT, and PLEK are commonly used software tools for lncRNA identification, and they were originally developed using human or other animal data for training. To improve the identification performance of plant lncRNA, we retrained the models of these tools using plant data sets and maintained their original settings. The code and data used for model retraining can be found at: https://github.com/xuechantian/lncRNA-Retraining.

Various metrics, including sensitivity, specificity, accuracy, precision, and the F1-score, were employed to evaluate the effectiveness of the lncRNA identification tools. These metrics were calculated using the following parameters: true positive (TP), which refers to the correct classification of long non-coding transcripts as LncRNA; true negative (TN), the correct classification of coding transcripts as mRNA; false positive (FP), the incorrect classification of coding transcripts as LncRNA; and false negative (FN), the incorrect classification of long non-coding transcripts as mRNA. Additionally, the ROC curve and the AUC were estimated to evaluate the performance of different classification models:


$$ \mathrm{Sensitivity}=\frac{\mathrm{TP}}{\mathrm{TP}+\mathrm{FN}} $$

\begin{align*} \mathrm{Specificity}=\frac{\mathrm{TN}}{\mathrm{FP}+\mathrm{TN}} \end{align*}

$$ \mathrm{Accuracy}=\frac{\mathrm{TP}+\mathrm{TN}}{\mathrm{TP}+\mathrm{FP}+\mathrm{TN}+\mathrm{FN}} $$

$$ \mathrm{Precision}=\frac{\mathrm{TP}}{\mathrm{TP}+\mathrm{FP}} $$

$$ \mathrm{F}1-\mathrm{score}=\frac{2\times \mathrm{Precision}\times \mathrm{Sensitivity}}{\mathrm{Precision}+\mathrm{Sensitivity}} $$

### A pipeline for lncRNA identification and characterization

We developed an lncRNA identification and characterization pipeline, Plant-LncPipe, to facilitate the discovery and characterization of novel plant lncRNAs (Fig. 5). Our Plant-LncPipe comprises three main modules for distinct tasks: (1) transcriptome alignment and transcriptome assembly; (2) lncRNA identification; and (3) lncRNA origin and classification. In module 1, RNA sequencing reads were mapped to the reference genome using HISAT2 [26], followed by transcript assembly with StringTie [27]. In module 2, a three-step approach was employed to identify lncRNAs in this pipeline. First, transcripts shorter than 200 bp and overlapping with known mRNAs were filtered out using the FEELnc_filter.pl module (—monoex = −1 and -s 200) of FEELnc [28]. Second, the protein coding potential of the remaining candidates was evaluated by an ensemble of the top two well-performing models, LncFinder-plant, and CPAT-plant. Lastly, the candidates were aligned to the UniProt protein database [29] using Diamond v2.0.2.140 [30] for further confirmation. Transcripts with alignment identity scores >80 and e-values <1e−05 were classified as potential coding transcripts and subsequently excluded from the predicted lncRNA set. Overall, this three-step approach provides a robust and reliable method for identifying lncRNAs from RNA sequencing reads with a high level of accuracy. In module 3, the identified lncRNAs were classified based on their genomic position using the FEELnc_classifier.pl module of FEELnc. The resulting categorized lncRNAs were then analyzed to determine their origin, specifically whether they arise from TEs. To identify TE-derived lncRNAs, the lncRNAs were intersected with TE annotations using the BEDTools program [31].

## Acknowledgements

We would like to acknowledge Dr Nathaniel Street from Umea Plant Science Centre (UPSC) for providing valuable language editing and insightful comments on this article. This research was supported by the National Key R&D Program of China (2022YFD2200103) and National Natural Science Foundation of China (32171816).

## Author contributions

J.F.M. conceived and designed the study; X.C.T., Z.Y.C., S.N., T.L.S., X.M.Y., Y.T.B., Z.C.L., H.Y.M., K.H.J., and W.Z. prepared the data and performed related analysis; X.C.T. wrote the manuscript; J.F.M. edited and improved the manuscript; and all authors approved the final manuscript.

## Data availability

Code and the data used for model construction can be found at: https://github.com/xuechantian/lncRNA-Retraining. The pipeline, Plant-LncPipe, is available at: https://github.com/xuechantian/Plant-LncRNA-pipline.

## Conflict of interest

The authors declare no competing interests.

## Supplementary data

Supplementary data are available at *Horticulture Research* online.

## Supplementary Material

Web_Material_uhae041

## References

[1] Mercer TR , Dinger ME, Mattick JS. Long non-coding RNAs: insights into functions. Nat Rev Genet. 2009;10:155–9 19188922 10.1038/nrg2521

[2] Ponting CP , Oliver PL, Reik W. Evolution and functions of long noncoding RNAs. Cell. 2009;136:629–41 19239885 10.1016/j.cell.2009.02.006

[3] Wierzbicki AT , Blevins T, Swiezewski S. Long noncoding RNAs in plants. Annu Rev Plant Biol. 2021;72:245–71 33752440 10.1146/annurev-arplant-093020-035446

[4] Qin T , Zhao H, Cui P. et al. A nucleus-localized long non-coding RNA enhances drought and salt stress tolerance. Plant Physiol. 2017;175:1321–36 28887353 10.1104/pp.17.00574 PMC5664461

[5] Derrien T , Johnson R, Bussotti G. et al. The GENCODE v7 catalog of human long noncoding RNAs: analysis of their gene structure, evolution, and expression. Genome Res. 2012;22:1775–89 22955988 10.1101/gr.132159.111 PMC3431493

[6] Kang Y-J , Yang D-C, Kong L. et al. CPC2: a fast and accurate coding potential calculator based on sequence intrinsic features. Nucleic Acids Res. 2017;45:W12–6 28521017 10.1093/nar/gkx428 PMC5793834

[7] Sun L , Luo H, Bu D. et al. Utilizing sequence intrinsic composition to classify protein-coding and long non-coding transcripts. Nucleic Acids Res. 2013;41:e166–6 23892401 10.1093/nar/gkt646 PMC3783192

[8] Wang L , Park HJ, Dasari S. et al. CPAT: Coding-Potential Assessment Tool using an alignment-free logistic regression model. Nucleic Acids Res. 2013;41:e74–4 23335781 10.1093/nar/gkt006 PMC3616698

[9] Li A , Zhang J, Zhou Z. PLEK: a tool for predicting long non-coding RNAs and messenger RNAs based on an improved k-mer scheme. BMC Bioinformatics. 2014;15:311 25239089 10.1186/1471-2105-15-311 PMC4177586

[10] Yang C , Yang L, Zhou M. et al. LncADeep: an ab initio lncRNA identification and functional annotation tool based on deep learning. Bioinformatics. 2018;34:3825–34 29850816 10.1093/bioinformatics/bty428

[11] Han S , Liang Y, Ma Q. et al. LncFinder: an integrated platform for long non-coding RNA identification utilizing sequence intrinsic composition, structural information and physicochemical property. Brief Bioinform. 2019;20:2009–27 30084867 10.1093/bib/bby065 PMC6954391

[12] Negri TC , Alves WAL, Bugatti PH. et al. Pattern recognition analysis on long noncoding RNAs: a tool for prediction in plants. Brief Bioinform. 2019;20:682–9 29697740 10.1093/bib/bby034

[13] Singh U , Khemka N, Rajkumar MS. et al. PLncPRO for prediction of long non-coding RNAs (lncRNAs) in plants and its application for discovery of abiotic stress-responsive lncRNAs in rice and chickpea. Nucleic Acids Res. 2017;45:e183 29036354 10.1093/nar/gkx866 PMC5727461

[14] Simopoulos CMA , Weretilnyk EA, Golding GB. Prediction of plant lncRNA by ensemble machine learning classifiers. BMC Genomics. 2018;19:316 29720103 10.1186/s12864-018-4665-2 PMC5930664

[15] Kern C , Wang Y, Chitwood J. et al. Genome-wide identification of tissue-specific long non-coding RNA in three farm animal species. BMC Genomics. 2018;19:684 30227846 10.1186/s12864-018-5037-7 PMC6145346

[16] Lv Y , Hu F, Zhou Y. et al. Maize transposable elements contribute to long non-coding RNAs that are regulatory hubs for abiotic stress response. BMC Genomics. 2019;20:864 31729949 10.1186/s12864-019-6245-5 PMC6858665

[17] Pedro DLF , Lorenzetti APR, Domingues DS. et al. PlaNC-TE: a comprehensive knowledgebase of non-coding RNAs and transposable elements in plants. Database. 2018;2018:bay078 30101318 10.1093/database/bay078 PMC6146122

[18] Jha UC , Nayyar H, Jha R. et al. Long non-coding RNAs: emerging players regulating plant abiotic stress response and adaptation. BMC Plant Biol. 2020;20:466 33046001 10.1186/s12870-020-02595-x PMC7549229

[19] Palos K , La Y, Railey CE. et al. Linking discoveries, mechanisms, and technologies to develop a clearer perspective on plant long noncoding RNAs. Plant Cell. 2023;35:1762–86 36738093 10.1093/plcell/koad027 PMC10226578

[20] Paytuví Gallart A , Hermoso Pulido A, Martínez A. et al. GREENC: a Wiki-based database of plant lncRNAs. Nucleic Acids Res. 2016;44:D1161–6 26578586 10.1093/nar/gkv1215 PMC4702861

[21] Jin J , Lu P, Xu Y. et al. PLncDB V2.0: a comprehensive encyclopedia of plant long noncoding RNAs. Nucleic Acids Res. 2021;49:D1489–95 33079992 10.1093/nar/gkaa910 PMC7778960

[22] Goodstein DM , Shu S, Howson R. et al. Phytozome: a comparative platform for green plant genomics. Nucleic Acids Res. 2012;40:D1178–86 22110026 10.1093/nar/gkr944 PMC3245001

[23] Szcześniak MW , Bryzghalov O, Ciomborowska-Basheer J. et al. CANTATAdb 2.0: expanding the collection of plant long noncoding RNAs. Methods Mol Biol. 2019;1933:415–29 30945201 10.1007/978-1-4939-9045-0_26

[24] Bolser D , Staines DM, Pritchard E. et al. Ensembl plants: integrating tools for visualizing, mining, and analyzing plant genomics data. Methods Mol Biol. 2016;1374:115–40 26519403 10.1007/978-1-4939-3167-5_6

[25] Li W , Godzik A. Cd-hit: a fast program for clustering and comparing large sets of protein or nucleotide sequences. Bioinformatics. 2006;22:1658–9 16731699 10.1093/bioinformatics/btl158

[26] Kim D , Langmead B, Salzberg SL. HISAT: a fast spliced aligner with low memory requirements. Nat Methods. 2015;12:357–60 25751142 10.1038/nmeth.3317 PMC4655817

[27] Pertea M , Pertea GM, Antonescu CM. et al. StringTie enables improved reconstruction of a transcriptome from RNA-seq reads. Nat Biotechnol. 2015;33:290–5 25690850 10.1038/nbt.3122 PMC4643835

[28] Wucher V , Legeai F, Hédan B. et al. FEELnc: a tool for long non-coding RNA annotation and its application to the dog transcriptome. Nucleic Acids Res. 2017;45:gkw1306–e57 10.1093/nar/gkw1306 PMC5416892 28053114

[29] Uniprot Consortium . UniProt: a worldwide hub of protein knowledge. Nucleic Acids Res. 2018;47:D506–15 10.1093/nar/gky1049 PMC6323992 30395287

[30] Buchfink B , Xie C, Huson DH. Fast and sensitive protein alignment using DIAMOND. Nat Methods. 2015;12:59–60 25402007 10.1038/nmeth.3176

[31] Quinlan AR , Hall IM. BEDTools: a flexible suite of utilities for comparing genomic features. Bioinformatics. 2010;26:841–2 20110278 10.1093/bioinformatics/btq033 PMC2832824

