# Supplementary material for: Plant-LncPipe: a computational pipeline providing significant improvement in plant lncRNA identification
Source: Hortic Res. 2024 Feb 8;11(4):uhae041. doi: 10.1093/hr/uhae041 (PMC11024640; doi:10.1093/hr/uhae041)
Supplement: Web_Material_uhae041 [file web_material_uhae041.zip › Revised Supporting Figure.docx]

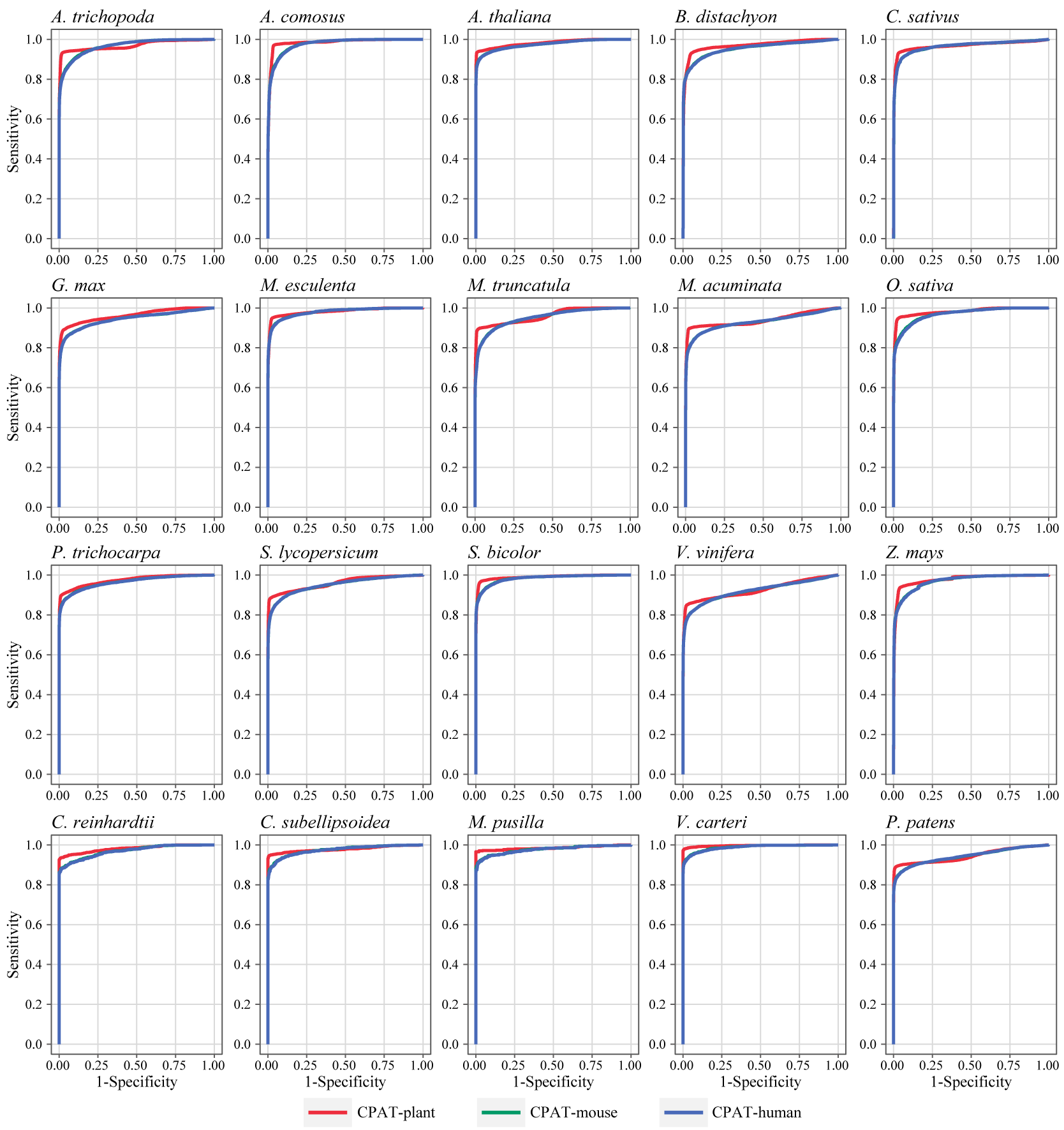


**Fig. S1.** ROC curves of the retrained models of CPAT (CPAT-plant) and their original models (CPAT-human and CPAT-mouse) on 20 plant datasets.

\
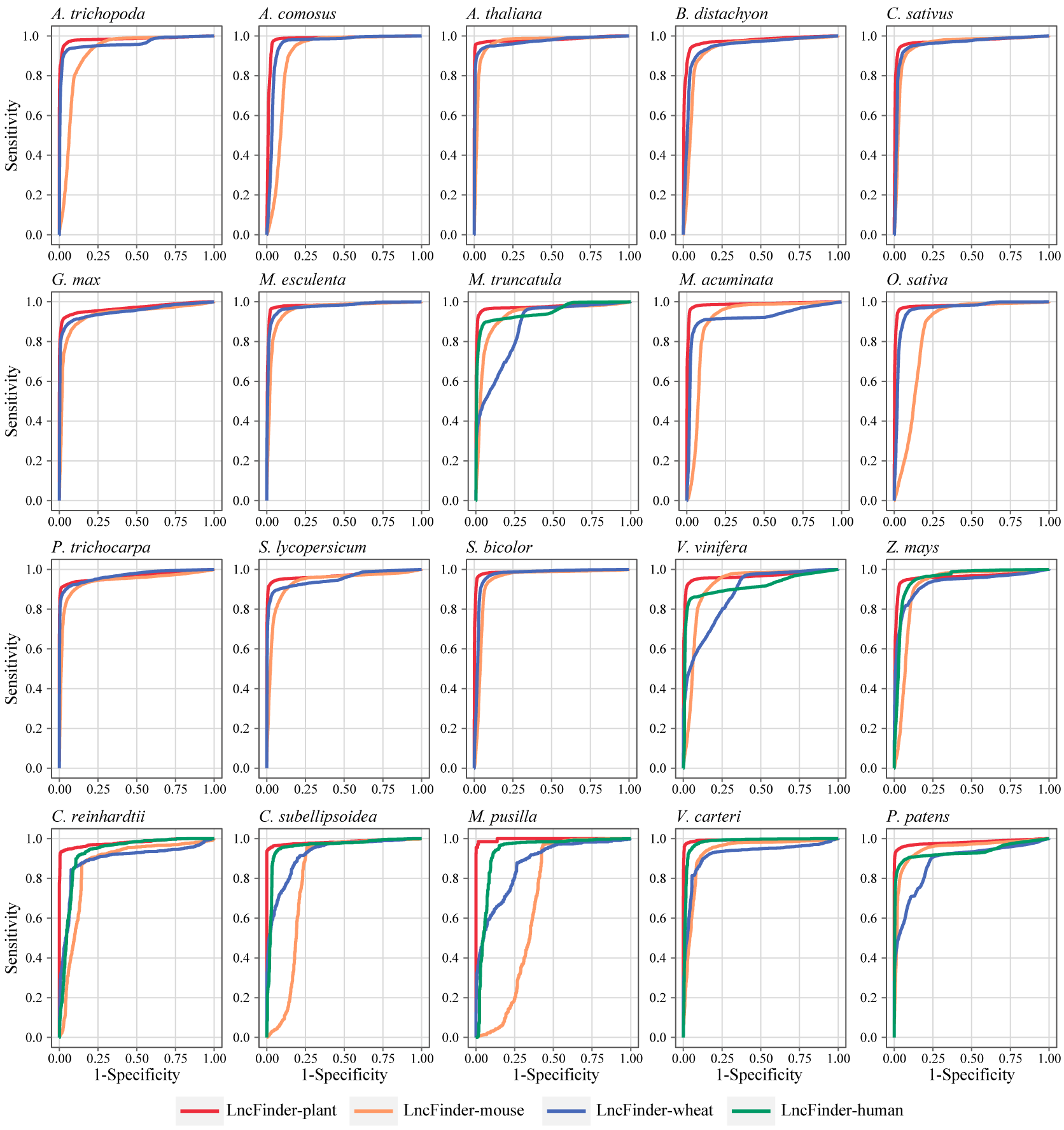


**Fig. S2.** ROC curves of the retrained models of LncFinder (LncFinder-plant) and their original models (LncFinder-human, LncFinder-mouse and LncFinder-wheat) on 20 plant datasets.


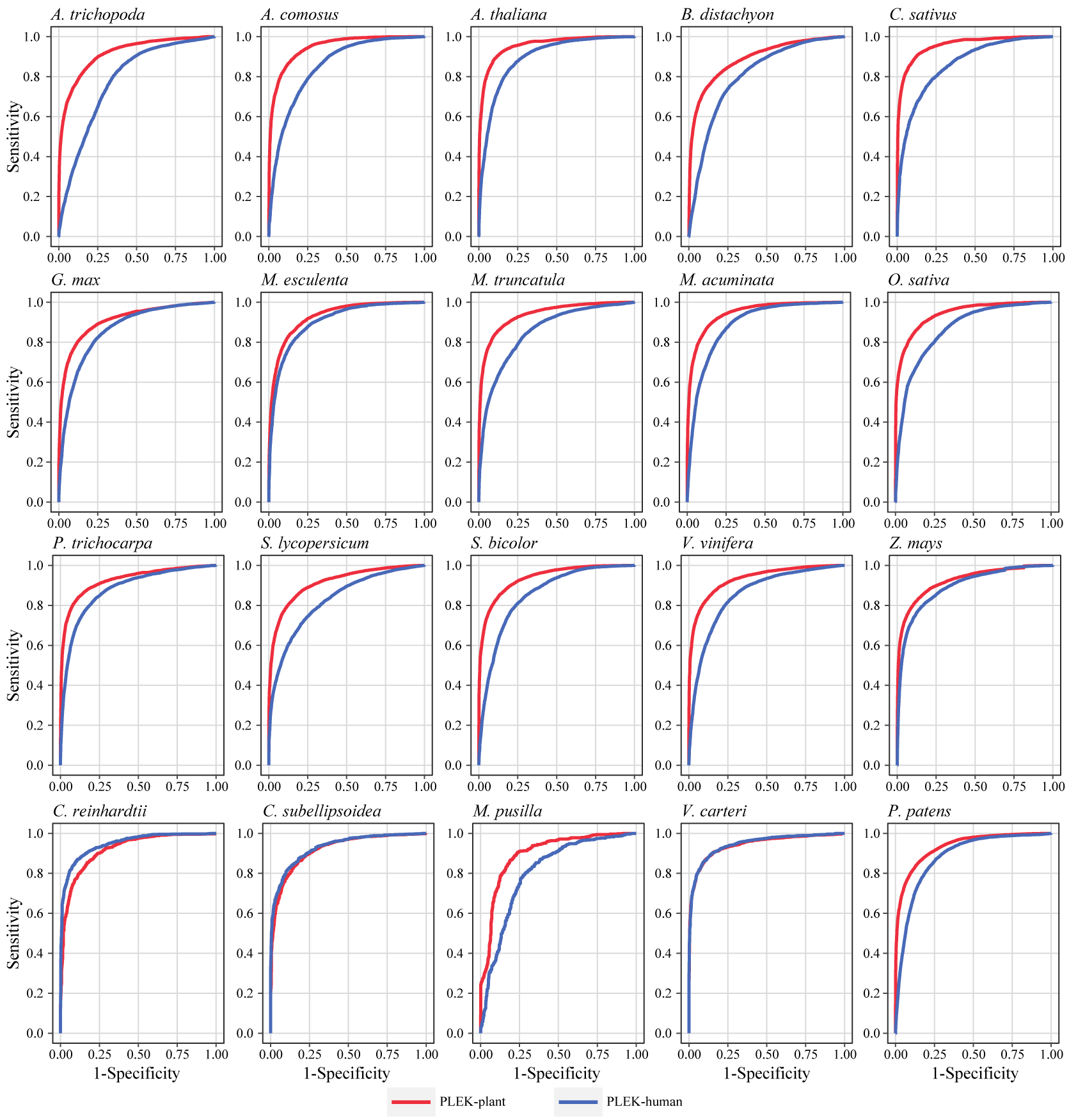


**Fig. S3.** ROC curves of the retrained models of PLEK (PLEK-plant) and their original models (PLEK-human) on 20 plant datasets.

**
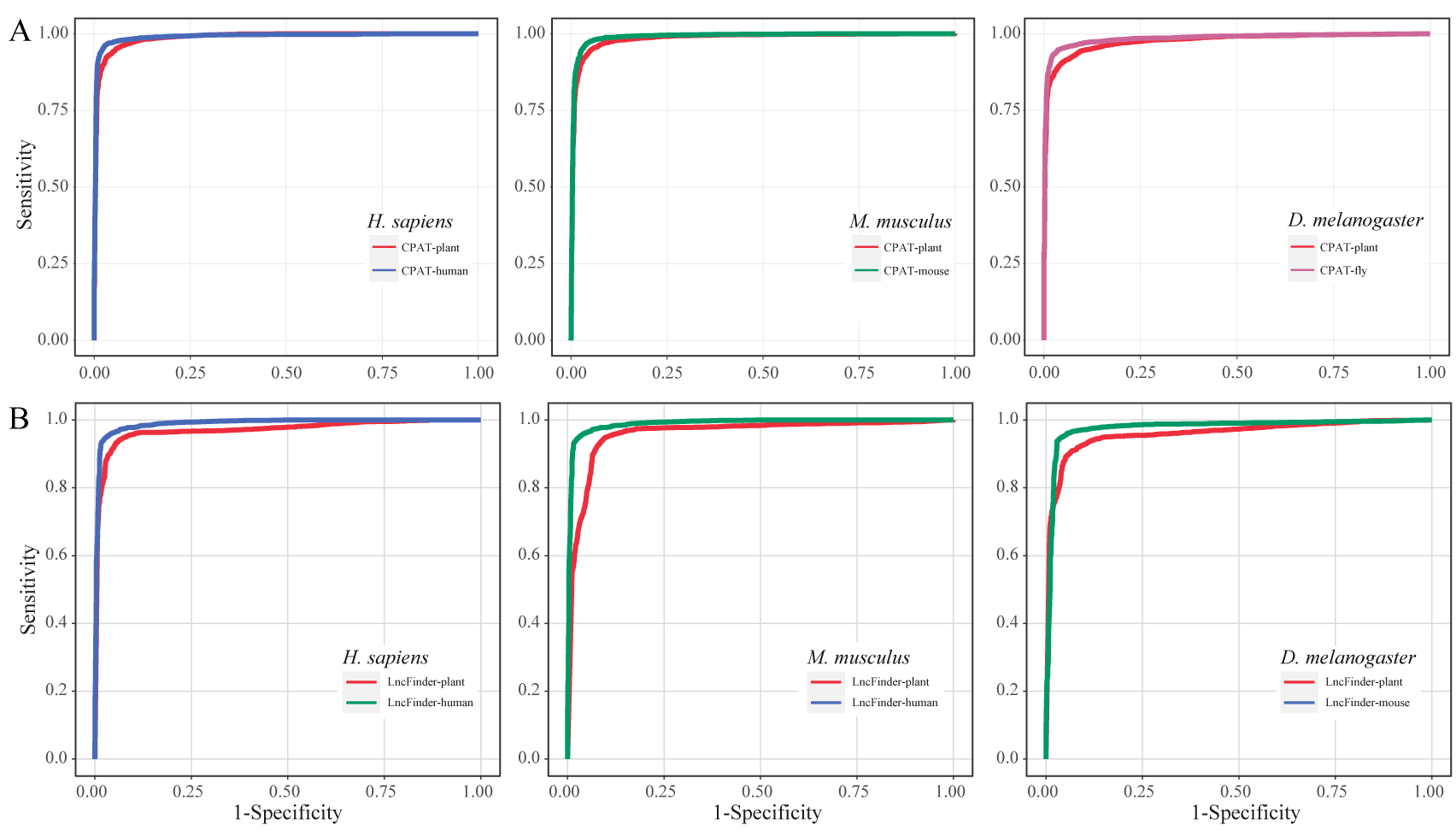
**

**Fig. S4.** ROC curves of the retrained and original models on datasets from three animal species. (A) ROC curves of the retrained (CPAT-plant) and the original models (CPAT-human and CPAT-mouse) of CPAT. (B) ROC curves of the retrained (LncFinder-plant) and the original models (LncFinder-human and LncFinder-mouse) of LncFinder.
