# Supplementary figures and images for: Plant-LncPipe: a computational pipeline providing significant improvement in plant lncRNA identification
Source: Hortic Res. 2024 Feb 8;11(4):uhae041. doi: 10.1093/hr/uhae041 (PMC11024640; doi:10.1093/hr/uhae041)

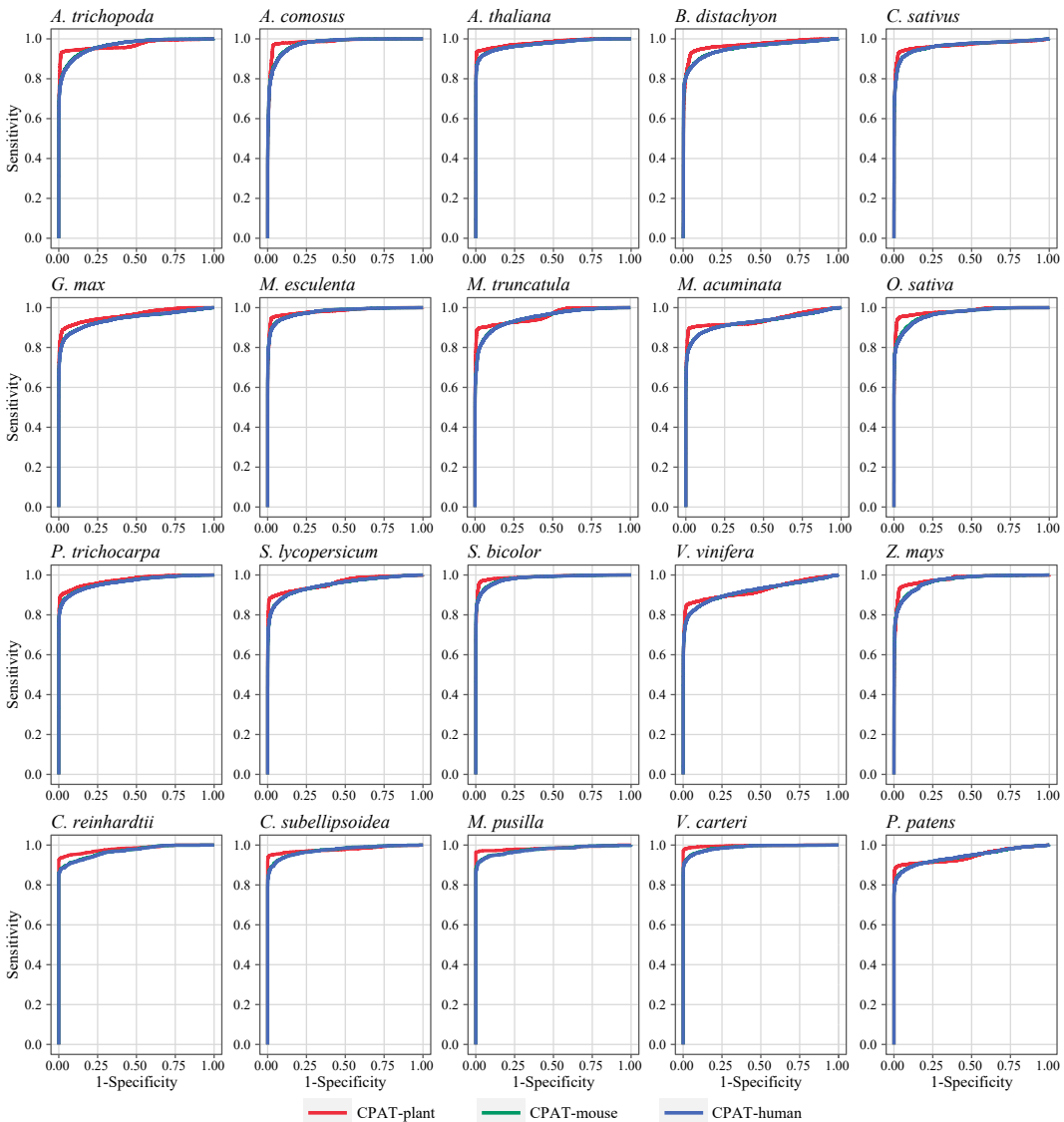

Supplement: Web_Material_uhae041 [file web_material_uhae041.zip › Fig. S1.pdf]

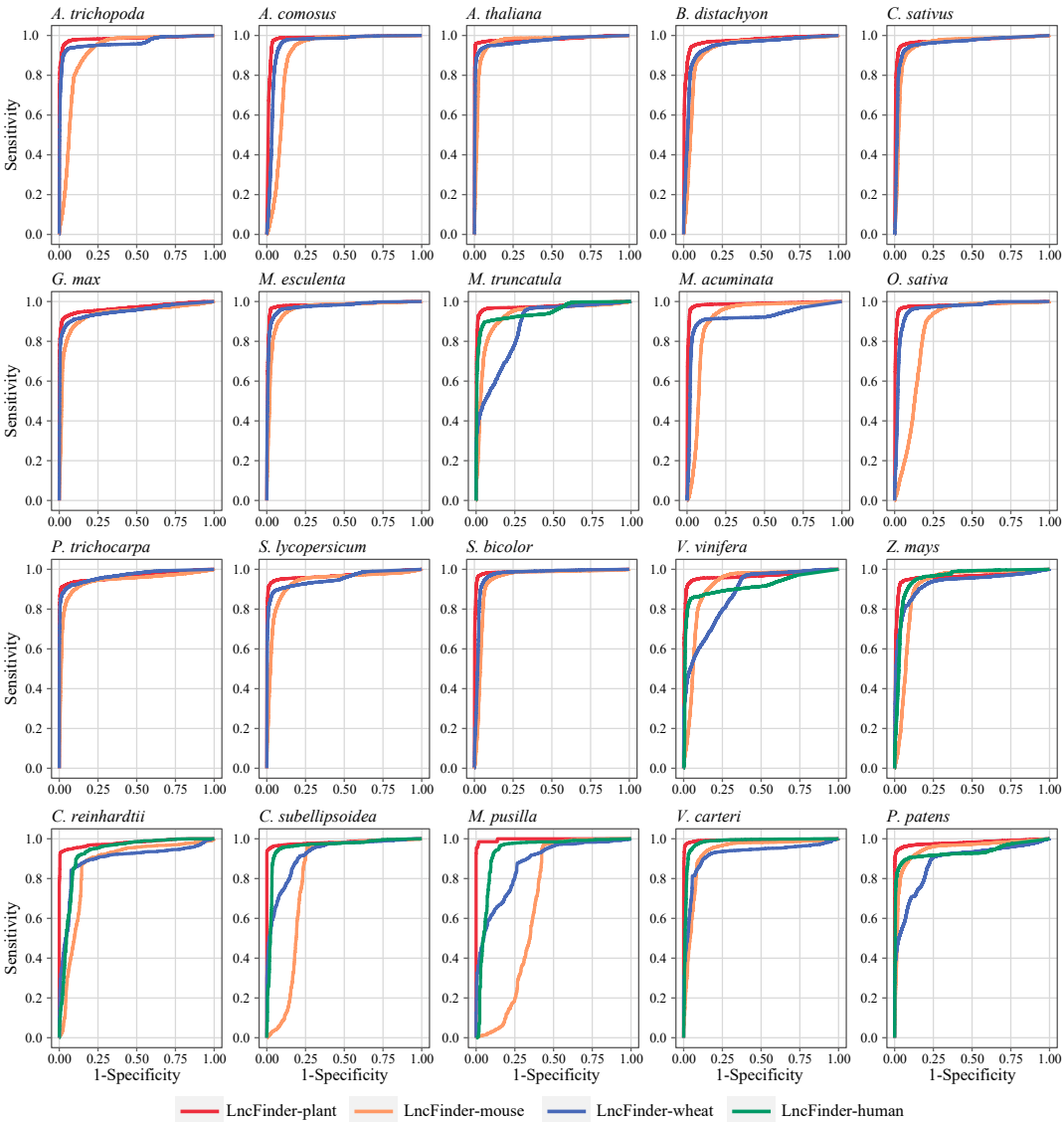

Supplement: Web_Material_uhae041 [file web_material_uhae041.zip › Fig. S2.pdf]

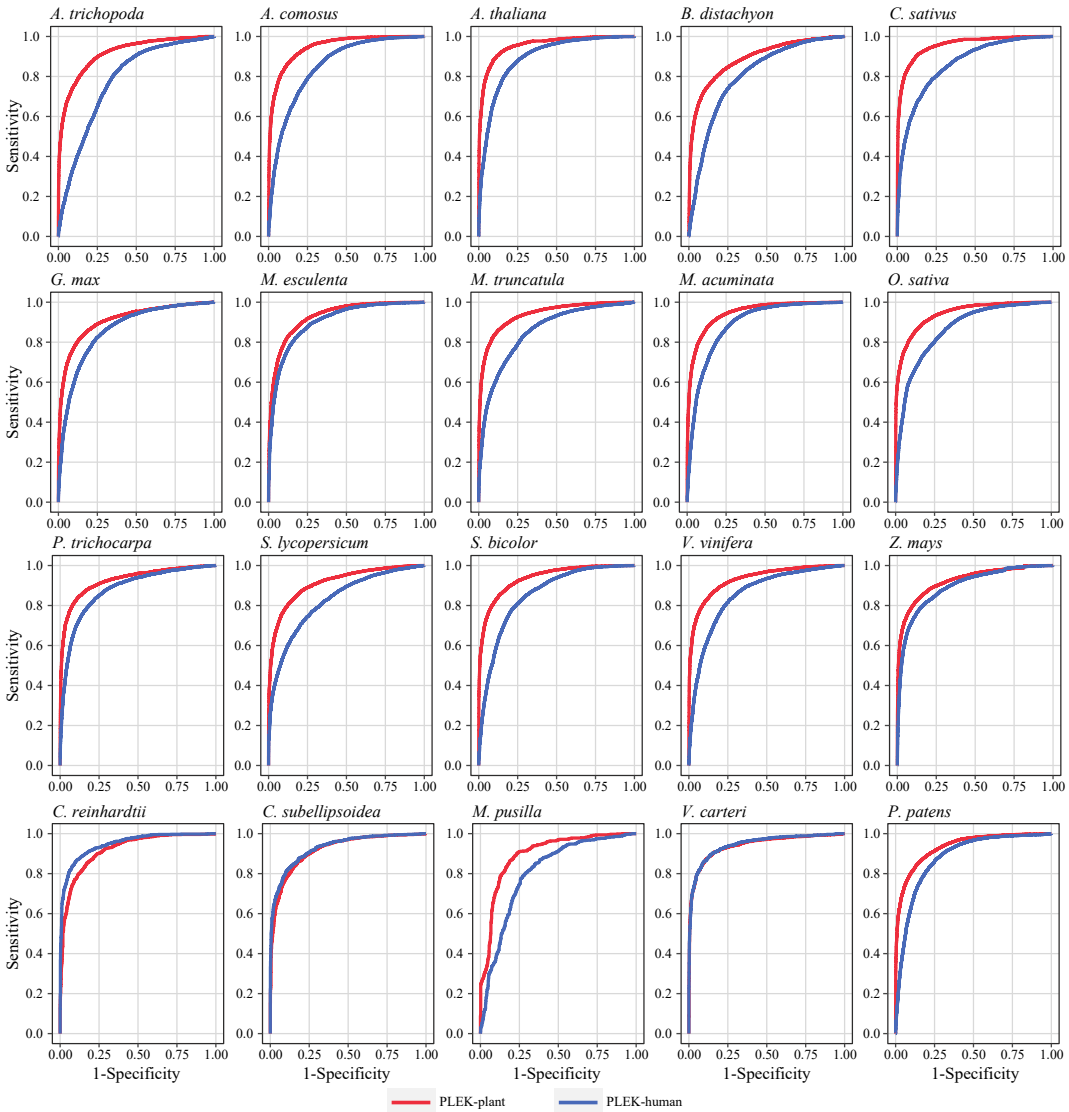

Supplement: Web_Material_uhae041 [file web_material_uhae041.zip › Fig. S3.pdf]

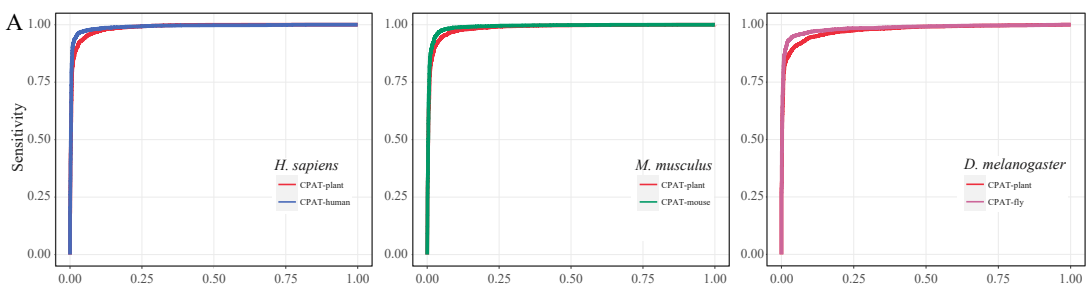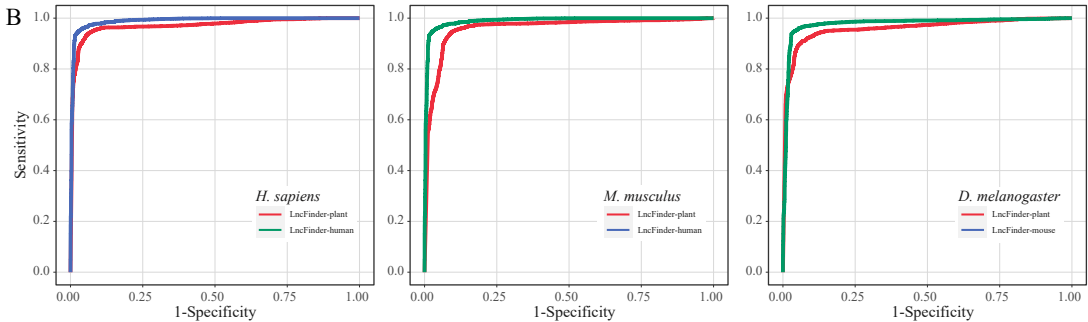

Supplement: Web_Material_uhae041 [file web_material_uhae041.zip › Fig. S4.pdf]
